# Supplementary material for: Bioinspired engineering of fusogen and targeting moiety equipped nanovesicles
Source: Nat Commun. 2023 Jun 8;14:3366. doi: 10.1038/s41467-023-39181-2 (PMC10250350; doi:10.1038/s41467-023-39181-2)
Supplement: Supplementary file 1 — Supplementary Information [file 41467_2023_39181_MOESM1_ESM.pdf]

Supplementary Information for

Bioinspired engineering of fusogen and targeting moiety equipped nanovesicles

Wang *et al.*

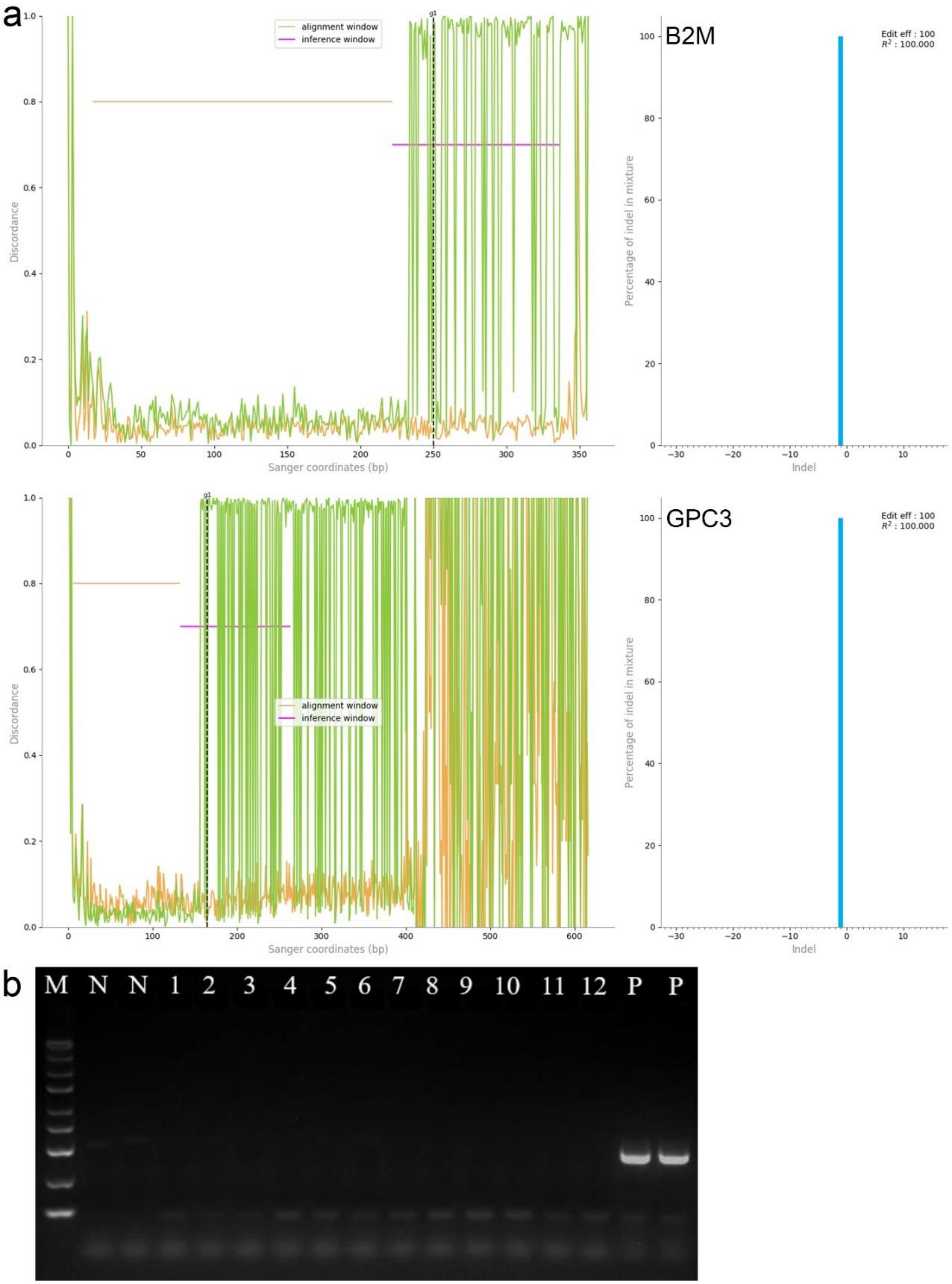

Supplementary Figure 1. Cell construction. (a) Indel distribution of double KO HEK293 cells. The discordance plot (left) details the level of alignment per base between the wild type (control) and the edited sample in the inference window (the region around the cute site). The Indel plot (right) displays the inferred distribution of indels in the entire edited population of genomes. Hovering over each bar of the Indel plot shows the size of the deletion, along with the percentage of genomes that contain it. (b) Mycoplasma detection. N: negative control, Lane 1-3: wild-type HEK293 cells, Lane 4-6: double KO HEK293 cells, Lane 7-9: eT-HEK293 cells, Lane 10-13: eFT-HEK293 cells, and P: positive control. Experiments were repeated thrice.

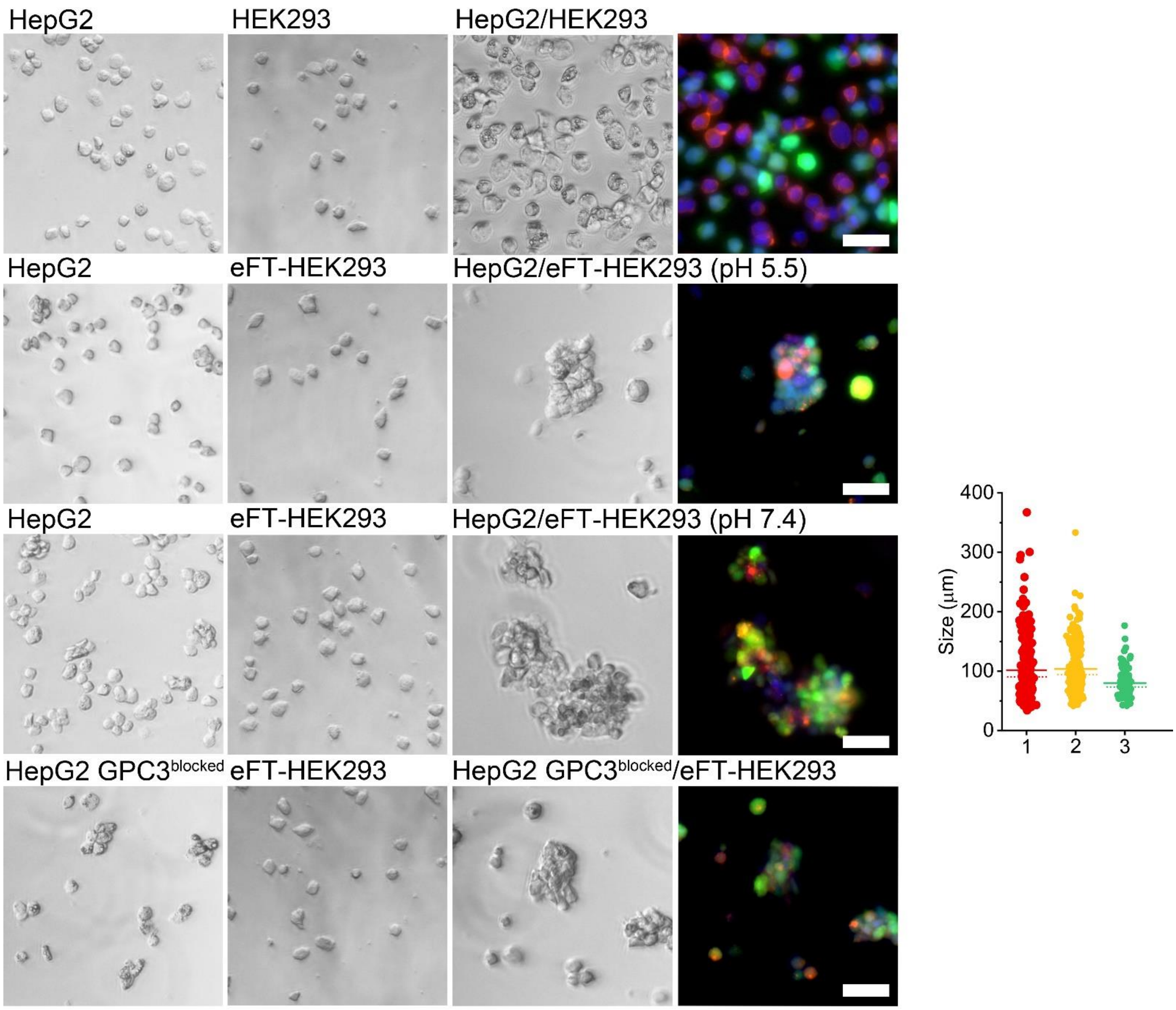

Supplementary Figure 2. Cell agglomeration. Equal amount of HepG2 cells and different PKH26 labeled HEK293 or eFT-HEK293 cells were thoroughly mixed and incubated in suspension at 37 °C for 3 h (Green: GFP, Red: PHK26 staining, and Blue: DAPI staining; scale bar is 40 µm). The geometric size distribution, mean size (solid line), and media size (dotted line) were measured and plotted (1: HepG2/eFT-HEK293 at pH 5.5, n=261; 2: HepG2/eFT-HEK293 at pH 5.5, n=246; and 3: HepG2 GPC3^{blocked}/eFT-HEK293, n=87; p<0.05, one-way ANOVA)

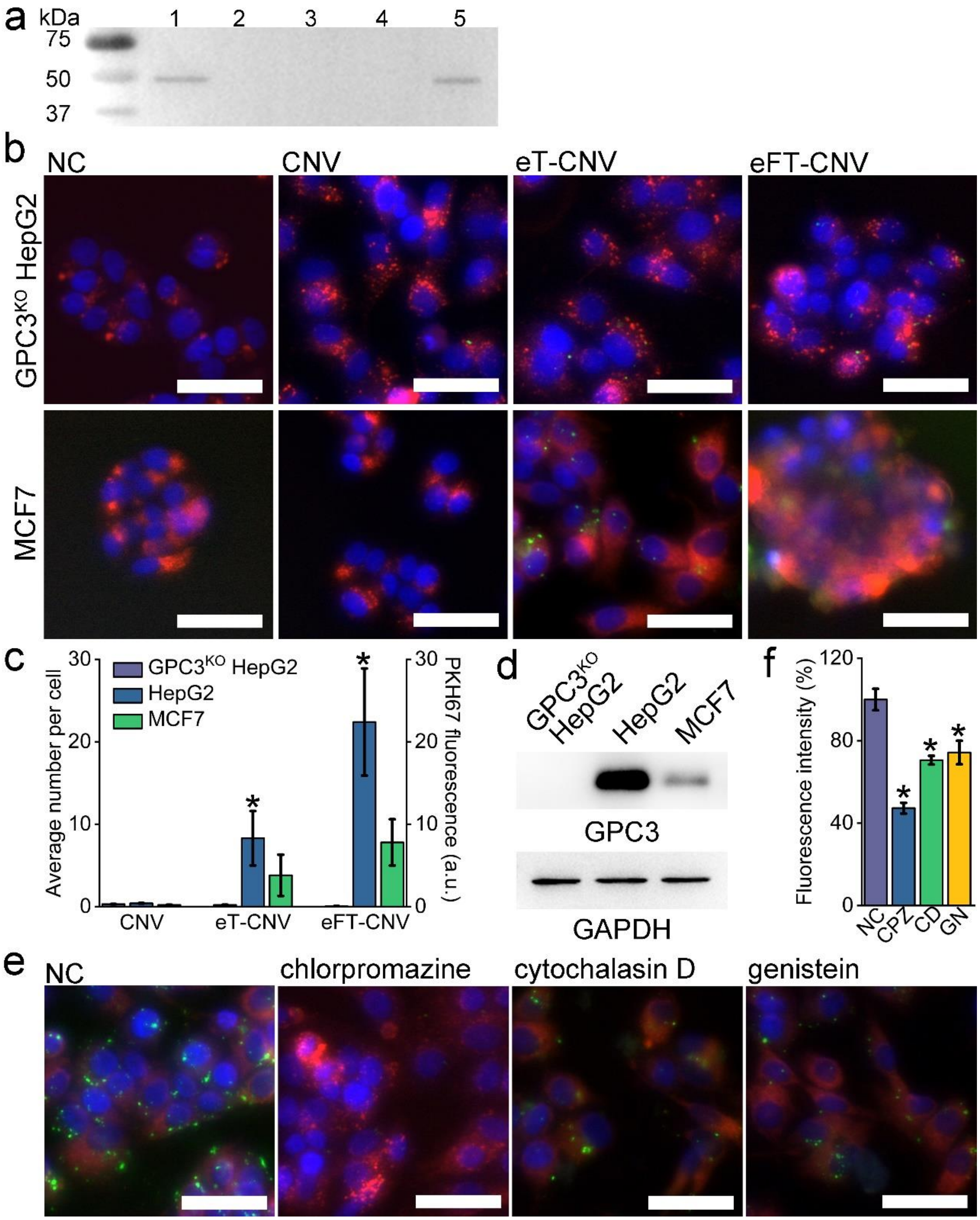

Supplementary Figure 3. Binding specificity of nanovesicles. (a) Western blot of E2 domain harboring a HA tag derived from (1) eFT-CNVs, (2) eT-CNVs, (3) CNVs, (4) HepG2 cells, and (5) eFT-CNVs treated HepG2 cells, respectively. Experiments were repeated thrice. (b) Approximately 1×10^8 PKH67 labeled CNVs, eT-CNVs, and eFT-CNVs treated GPC3^{KO} HepG2 cells or MCF7 cells for 30 min (NC: negative control, Green: PKH67 dye staining, Red: Lysoview 594 staining, and Blue: DAPI staining; scale bar is 40 µm). (c) Quantitative data of average number of nanovesicles and average PKH67 fluorescence intensity (n=200; $p<0.001$, one-way ANOVA). (d) Western blot of GPC3 protein expression in GPC3^{KO} HepG2 cells and MCF7 cells. (e) Images of eT-CNVs uptake into HepG2 cells at 37 °C pre-incubated with chlorpromazine (CPZ), cytochalasin D (CD), and genistein (GN) for 30 min (scale bar is 40 µm). (f) Quantitative data of average fluorescence intensity (n=200; $p<0.05$, one-way ANOVA).

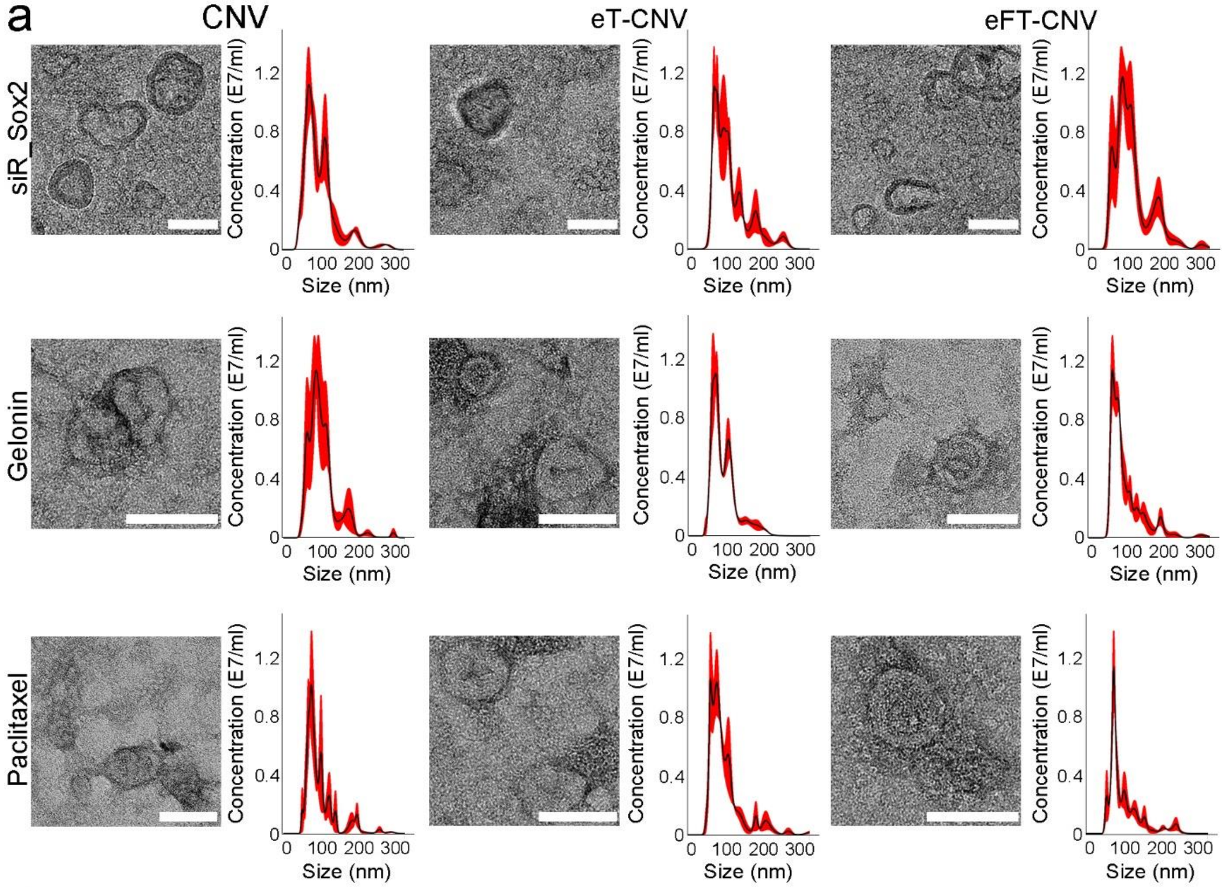

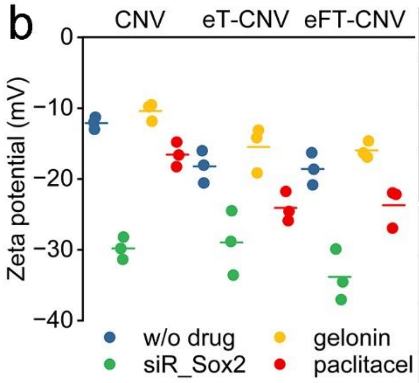

	empty	siR-Sox2	gelonin	paclitaxel
CNV (nm)	114.9	140.8	136.9	123.6
eT-CNV (nm)	124.3	137.4	142.4	135.5
eFT-CNV (nm)	120.9	146.6	155.9	131.1

	empty	siR-Sox2	gelonin	paclitaxel
CNV (mV)	-12.1	-29.8	-10.4	-16.5
eT-CNV (mV)	-18.2	-28.9	-15.5	-24.1
eFT-CNV (mV)	-18.6	-33.8	-15.9	-23.7

Supplementary Figure 4. Physical characterization of drug-loaded nanovesicles. (a) Morphology and size distribution of drug-loaded nanovesicles (scale bar is 100 nm). Experiments were repeated thrice. (b) Changes of zeta potential of nanovesicles after drug loading (n=3).

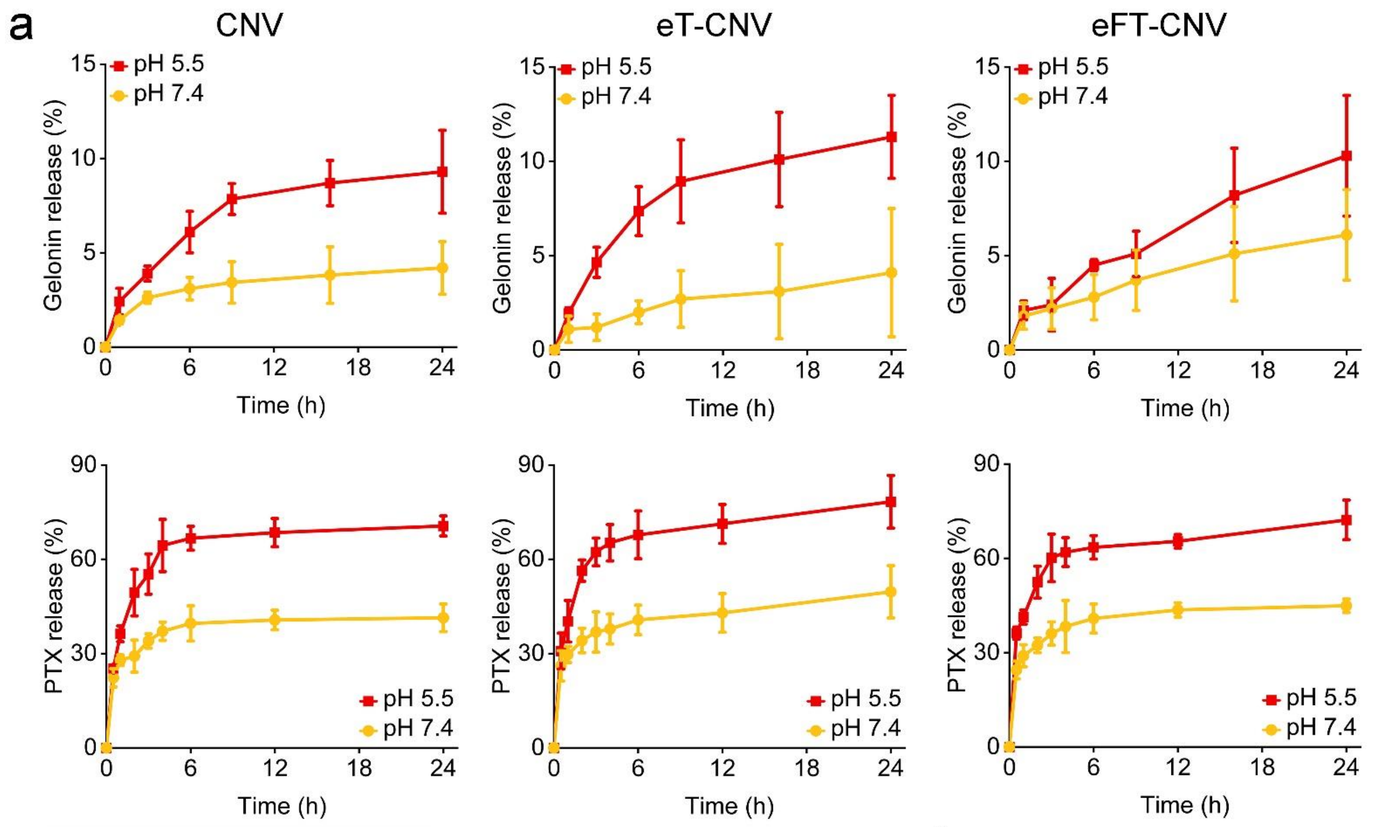

	pH 5.5	pH 7.4
	gelonin	
CNV	9.3%	4.2%
eT-CNV	11.3%	4.1%
eFT-CNV	10.3%	6.1%
	paclitaxel	
CNV	70.6%	41.4%
eT-CNV	77.8%	49.8%
eFT-CNV	73.2%	45.3%

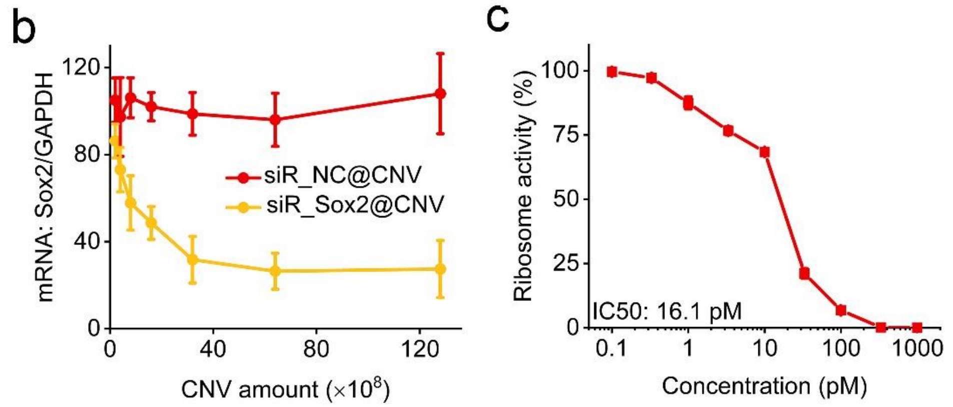

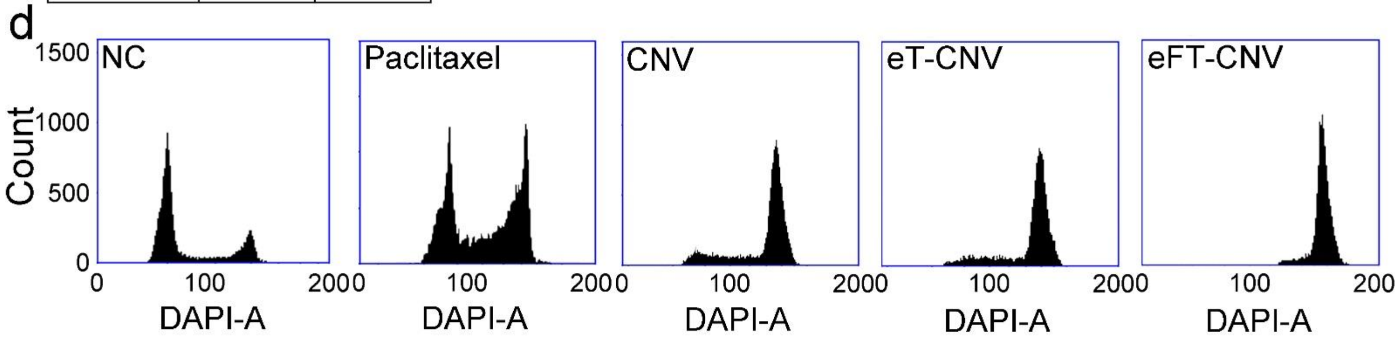

Supplementary Figure 5. Chemical characterization of drug-loaded nanovesicles. (a) *In vitro* release of gelonin and paclitaxel from CNVs, eT-CNVs, and eFT-CNVs at 37°C in either pH5.5 or pH 7.4 buffer, respectively, and quantitative data (n=5). (b) Verification of the treatment effect of siR-Sox2 in comparison to mock siRNA (n=5). (c) Ribosome-inactivating protein activity of free gelonin measured by using the cell-free Rabbit reticulocyte lysate assay (n=6). (d) The cell cycle of HepG cells treated with 200 nM free paclitaxel or paclitaxel equivalent in nanovesicles for 24 h, respectively.

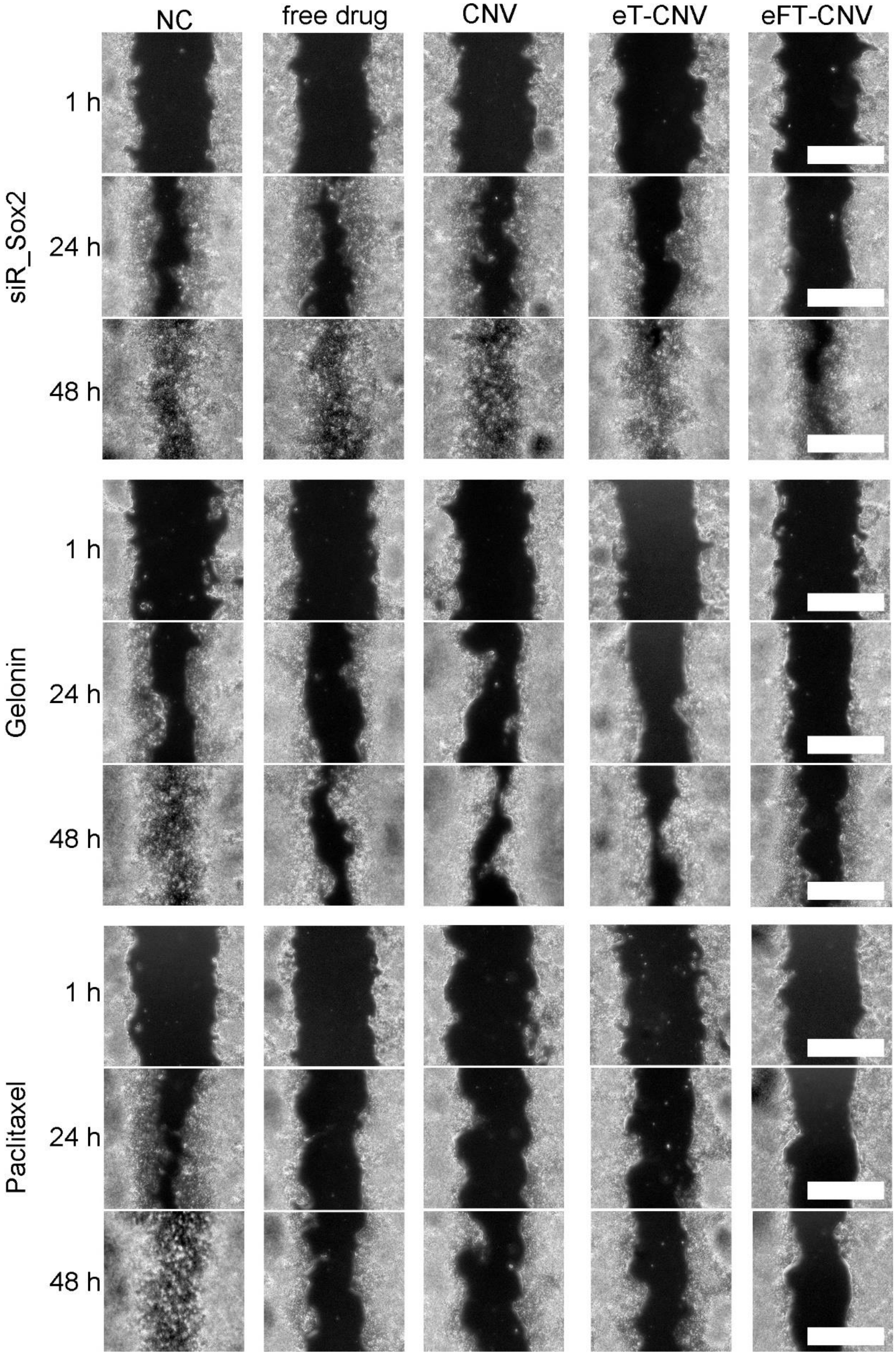

Supplementary Figure 6. Migration of treated HepG2 cells. Wound healing assay of HepG2 cells treated with free drug or drug-loaded nanovesicles. Migration was assessed at 1, 24, and 48 h after wounding (scale bar is 500 µm).

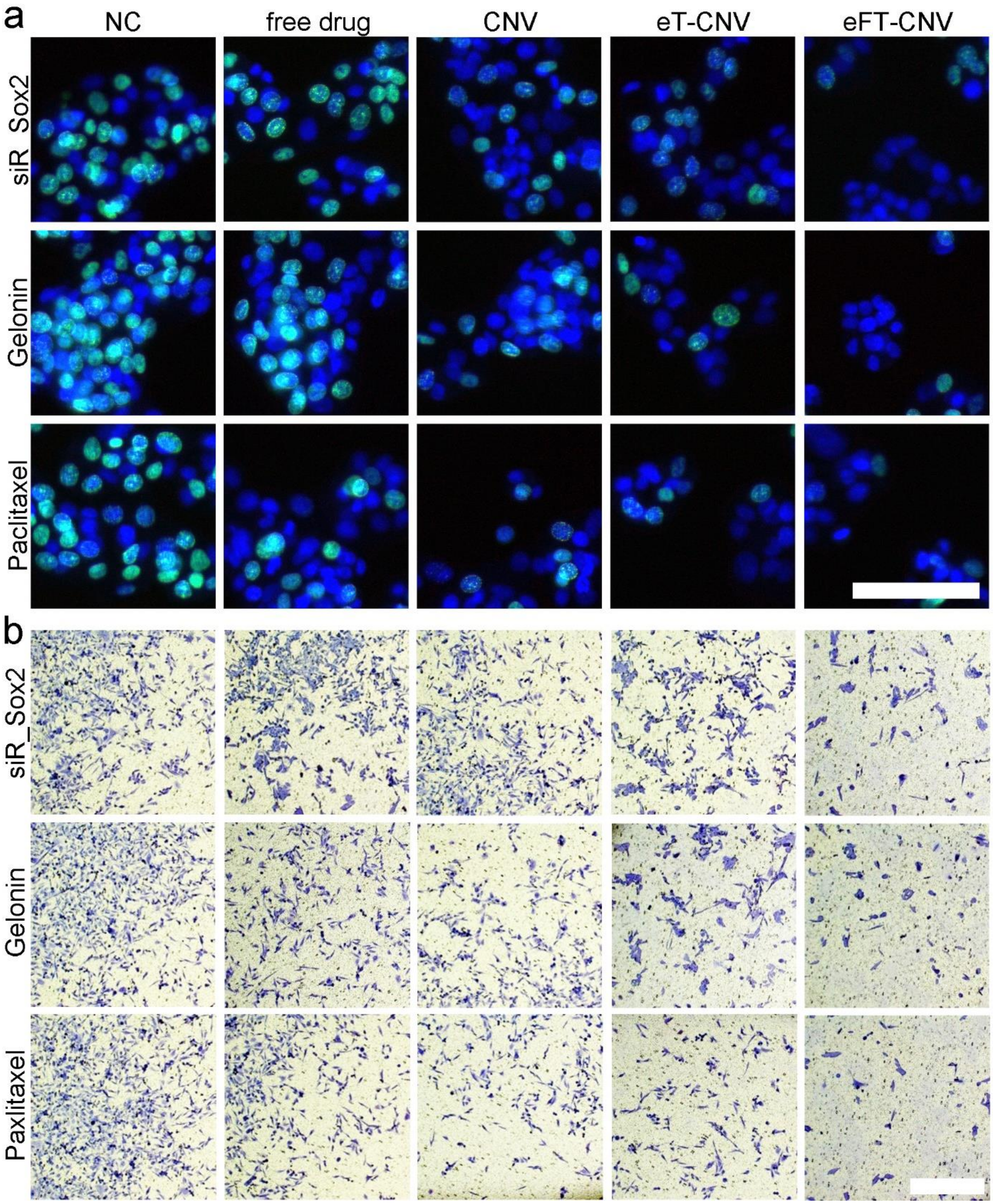

Supplementary Figure 7. Proliferation and invasion of treated HepG2 cells. (a) Fluorescence images of HepG2 cells treated with the free drug and drug-loaded nanovesicles (Green: EdU staining and Blue: DAPI staining; scale bar is 40 μm). Experiments were repeated thrice. (b) Optical images of invaded HepG2 cells treated with free drug or drug-loaded nanovesicles (Blue: crystal violet staining; scale bar is 400 μm). Experiments were repeated thrice.

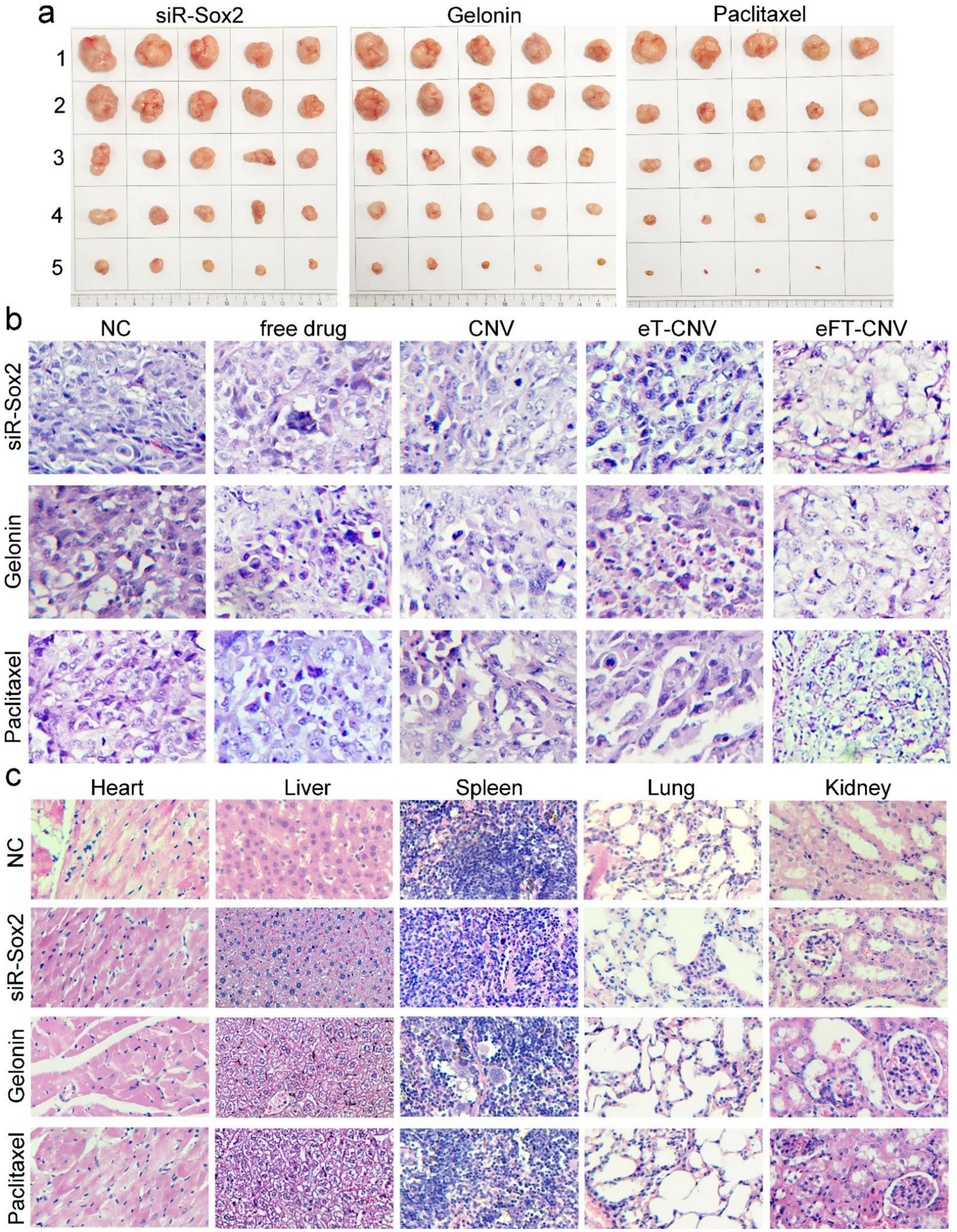

Supplementary Figure 8. Tumor treatment in vivo. (a) Tumor tissues obtained from each group of euthanized mice 28 days after administration of (1) PBS, (2) free drug, (3) drug-loaded CNVs, (4) drug-loaded eT-CNVs, and (5) drug-loaded eFT-CNVs, respectively. (b) Typical histologic section with HE

staining of tumors in each group. Experiments were repeated ten times. (c) HE staining of major organs in groups of PBS and paclitaxel-loaded eFT-CNVs. Experiments were repeated ten times.

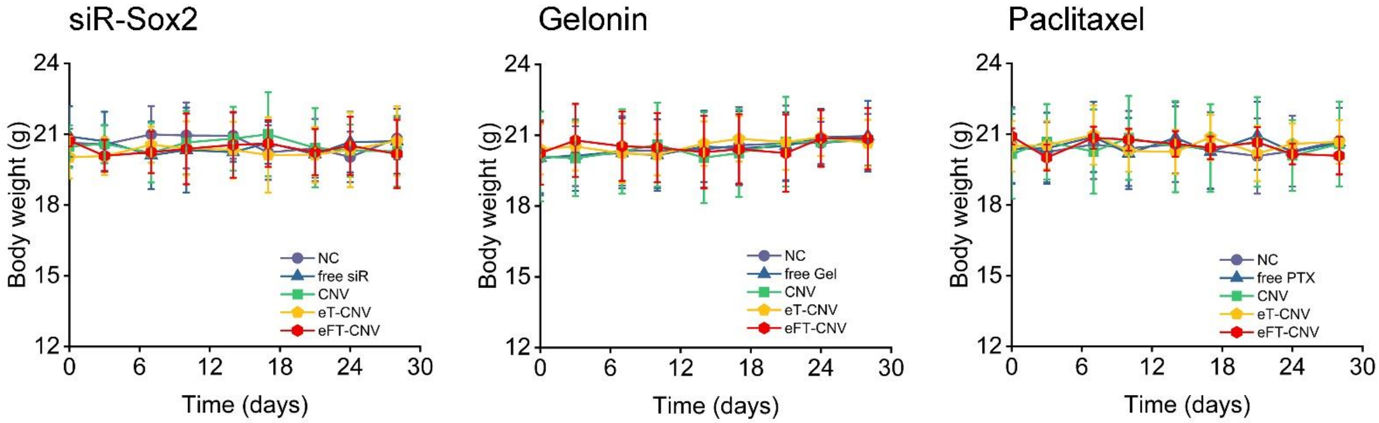

Supplementary Figure 9. Tumor treatment *in vivo*. Average mass of mice from each group without significant difference (n = 5).

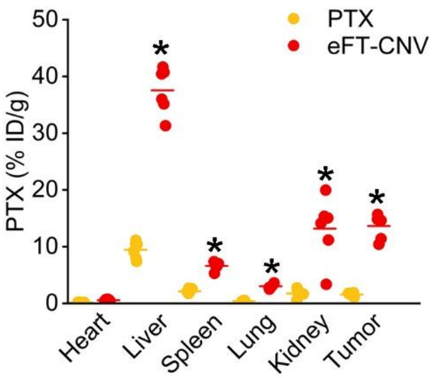

Supplementary Figure 10. Biodistribution of free paclitaxel and paclitaxel released from eFT-CNVs at 24 h post injection (n=6; p<0.05, two-way t-test).
