# Supplementary material for: Bioinspired engineering of fusogen and targeting moiety equipped nanovesicles
Source: Nat Commun. 2023 Jun 8;14:3366. doi: 10.1038/s41467-023-39181-2 (PMC10250350; doi:10.1038/s41467-023-39181-2)
Supplement: Supplementary file 3 — Reporting Summary [file 41467_2023_39181_MOESM3_ESM.pdf]

nature portfolio

Corresponding author(s): Yuan Wan

Last updated by author(s): May 10, 2023

Reporting Summary

Nature Portfolio wishes to improve the reproducibility of the work that we publish. This form provides structure for consistency and transparency in reporting. For further information on Nature Portfolio policies, see our Editorial Policies and the Editorial Policy Checklist.

Statistics

For all statistical analyses, confirm that the following items are present in the figure legend, table legend, main text, or Methods section.

n/a	Confirmed	
☐	☒	The exact sample size (*n*) for each experimental group/condition, given as a discrete number and unit of measurement
☐	☒	A statement on whether measurements were taken from distinct samples or whether the same sample was measured repeatedly
☐	☒	The statistical test(s) used AND whether they are one- or two-sided<br>*Only common tests should be described solely by name; describe more complex techniques in the Methods section.*
☐	☒	A description of all covariates tested
☐	☒	A description of any assumptions or corrections, such as tests of normality and adjustment for multiple comparisons
☐	☒	A full description of the statistical parameters including central tendency (e.g. means) or other basic estimates (e.g. regression coefficient) AND variation (e.g. standard deviation) or associated estimates of uncertainty (e.g. confidence intervals)
☐	☒	For null hypothesis testing, the test statistic (e.g. *F*, *t*, *r*) with confidence intervals, effect sizes, degrees of freedom and *P* value noted<br>*Give P values as exact values whenever suitable.*
☒	☐	For Bayesian analysis, information on the choice of priors and Markov chain Monte Carlo settings
☒	☐	For hierarchical and complex designs, identification of the appropriate level for tests and full reporting of outcomes
☒	☐	Estimates of effect sizes (e.g. Cohen's *d*, Pearson's *r*), indicating how they were calculated

Our web collection on statistics for biologists contains articles on many of the points above.

Software and code

Policy information about availability of computer code

Data collection	software that comes with Nikon NIS Elements 5.21.03, BD CFlow, Spectrum Compact Capillary Electrophoresis System v6138080-09, Flowjo 10.4, FEI Tecnai transmission electron microscope v2.0, AzureSpot Pro v2, Magellan Pro v7.3, Bio-Rad C1000, Vanquish v1.61
Data analysis	originlab2018, Flowjo 10.4, ImageJ 1.53t

For manuscripts utilizing custom algorithms or software that are central to the research but not yet described in published literature, software must be made available to editors and reviewers. We strongly encourage code deposition in a community repository (e.g. GitHub). See the Nature Portfolio guidelines for submitting code & software for further information.

Data

Policy information about availability of data

All manuscripts must include a data availability statement. This statement should provide the following information, where applicable:

- Accession codes, unique identifiers, or web links for publicly available datasets
- A description of any restrictions on data availability
- For clinical datasets or third party data, please ensure that the statement adheres to our policy

All relevant data of this study were presented in the paper and Supplementary Information file. Additional information and unique biological materials can be requested from the corresponding author upon reasonable request. Further details are described within the Supplementary Information file that includes

characterization of cells and nanovesicles, cell assays data, and animal study data. Custom code or mathematical algorithm was not involved in this study.

Human research participants

Policy information about studies involving human research participants and Sex and Gender in Research.

Reporting on sex and gender	n.a
Population characteristics	n.a
Recruitment	n.a
Ethics oversight	n.a

Note that full information on the approval of the study protocol must also be provided in the manuscript.

Field-specific reporting

Please select the one below that is the best fit for your research. If you are not sure, read the appropriate sections before making your selection.

☒ Life sciences ☐ Behavioural & social sciences ☐ Ecological, evolutionary & environmental sciences

For a reference copy of the document with all sections, see nature.com/documents/nr-reporting-summary-flat.pdf

Life sciences study design

All studies must disclose on these points even when the disclosure is negative.

Sample size	At least five biological replicates were used to satisfy t-test or ANOVA test. To get more reliable results and statistical significance, 7-200 replicates were used in few assays.
Data exclusions	No data were excluded from the analyses
Replication	3-10 times, depending on the number of treatments, availability of material, experimental unit, and expenses. All attempts at replication were successful.
Randomization	Mice were then randomly divided into 5 groups. Fluorescence images were randomly taken from substrates. Random sampling was strictly executed in whole study.
Blinding	n.a.; not involved. The operator must know which were the experimental groups.

Reporting for specific materials, systems and methods

We require information from authors about some types of materials, experimental systems and methods used in many studies. Here, indicate whether each material, system or method listed is relevant to your study. If you are not sure if a list item applies to your research, read the appropriate section before selecting a response.

Materials & experimental systems

n/a	Involved in the study	
☐	☒	Antibodies
☐	☒	Eukaryotic cell lines
☒	☐	Palaeontology and archaeology
☐	☒	Animals and other organisms
☒	☐	Clinical data
☒	☐	Dual use research of concern

Methods

n/a	Involved in the study	
☒	☐	ChIP-seq
☐	☒	Flow cytometry
☒	☐	MRI-based neuroimaging

Antibodies

Antibodies used	B2M: sc-13565, GPC3: sc-390587, GAPDH: sc-32233, HA: sc-7392, CD81: sc-166029, TSG101: sc-7964, HA fab fragment: H153-70A12 ordered from Creative Biolabs;

Validation	The specificity of the antibodies employed in this study were validated by immunoblot analysis. Appropriate positive and negative controls were included in every experiment. To check for antibody specificity, we used knockout cell lines (for protein of interest) as a control and ensure the right size bands disappear in those cells. In addition, we monitored the Antibody Registry database (http://antibodyregistry.org/). Additional validation files can be found through https://datasheets.scbt.com/sc-13565.pdf (B2M), https://datasheets.scbt.com/sc-390587.pdf (GPC3), https://datasheets.scbt.com/sc-32233.pdf (GAPDH), https://datasheets.scbt.com/sc-7392.pdf (HA), https://datasheets.scbt.com/sc-166029.pdf (CD81), https://datasheets.scbt.com/sc-7964.pdf (TSG101), and https://onlinelibrary.wiley.com/doi/abs/10.1111/j.1365-3083.1993.tb01676.x (HA fab fragment).

Eukaryotic cell lines

Policy information about cell lines and Sex and Gender in Research

Cell line source(s)	HEK293, HepG2, MCF7 were ordered from ATCC
Authentication	All cell lines were authenticated using the short tandem repeat (STR) profiling (Genetica).
Mycoplasma contamination	All cell lines were periodically tested for mycoplasma contamination using e-Myco Plus kit (iNtRON Biotechnology). None of the cell lines were contaminated.
Commonly misidentified lines (See ICLAC register)	no commonly misidentified cell lines were used in this study.

Animals and other research organisms

Policy information about studies involving animals; ARRIVE guidelines recommended for reporting animal research, and Sex and Gender in Research

Laboratory animals	BALB/c mice, ~18-22 g, 6 weeks; The housing conditions for the mice were as follows: 12:12 h dark/light cycle, ambient temperature of 22±1 °C, and ~55% of humidity
Wild animals	no wild animals were used in this study
Reporting on sex	GPC3 overexpression across a broad spectrum of tumor types, and our findings apply to both male and female sex. Therefore, both male and female mice were used for the in vivo experiments described in the manuscript.
Field-collected samples	no field collected samples were used in this study.
Ethics oversight	All animal experiments were approved by and performed by guidelines from the Institutional Animal Care and Use Committee (IACUC) of the Model Animal Research Center of the Second Hospital of Nanjing.

Note that full information on the approval of the study protocol must also be provided in the manuscript.

Flow Cytometry

Plots

Confirm that:

☒ The axis labels state the marker and fluorochrome used (e.g. CD4-FITC).

☒ The axis scales are clearly visible. Include numbers along axes only for bottom left plot of group (a 'group' is an analysis of identical markers).

☒ All plots are contour plots with outliers or pseudocolor plots.

☒ A numerical value for number of cells or percentage (with statistics) is provided.

Methodology

Sample preparation	The constructed HEK293 cells were cultured with indicated agents in medium supplemented with 0.1% FBS for 24 h at 37 °C. The cells were labeled with anti-HA antibody (1:200, sc-7392, Santa Cruz) as an indicator of successful expression of anti-GPC3 scFv on cell membranes. The labeled cells were characterized with flow cytometry.
Instrument	BD Accuri C6
Software	BD CFlow, Flowjo 10.4
Cell population abundance	NA. samples were not sorted.

Gating strategy

cells were first gated to exclude cell debris and aggregates based on FSC/SSC. Then cells stained with anti-HA antibody were used to determine the boundary between "negative" and "positive" cells. this boundary was used to identify cells overexpressing anti-GPC3 scFv on membranes.

☒ Tick this box to confirm that a figure exemplifying the gating strategy is provided in the Supplementary Information.
